# Ictal Modulation of Cardiac Repolarization, but Not of Heart Rate, Is Lateralized in Mesial Temporal Lobe Epilepsy

**DOI:** 10.1371/journal.pone.0064765

**Published:** 2013-05-31

**Authors:** Rainer Surges, Arthur Jordan, Christian E. Elger

**Affiliations:** Department of Epileptology, University Hospital Bonn, Bonn, Germany; University of Adelaide, Australia

## Abstract

**Objectives:**

Human and animal studies provided controversial data on asymmetric cortical representation of cardiac function, which may partially be due to different study designs and inter-individual variability. Here, we investigated whether seizure-related changes in heart rate (HR) and cardiac repolarization depend on the side of seizure-activity in people with mesial temporal lobe epilepsy (mTLE).

**Methods:**

To account for inter-individual variability, EEG and ECG data were reviewed from patients with medically refractory mTLE undergoing pre-surgical video-EEG telemetry with at least 2 seizures arising from each hippocampus as assessed by bilateral hippocampal depths electrodes. RR and QT intervals were determined at different timepoints using a one-lead ECG. QT intervals were corrected for HR (QTc) using 4 established formulas.

**Results:**

Eighty-two seizures of 15 patients were analyzed. HR increased by ∼30% during hippocampal activity irrespective of the side (p = 0.411). QTc intervals were lengthened to a significantly greater extent during left hippocampal seizures (e.g. difference of QT intervals between preictal and ictal state using Bazett’s formula; left side 32.0±5.3 ms, right side 15.6±7.7 ms; p = 0.016). Abnormal QTc prolongation occurred in 7 of 41 left hippocampal seizures of 4 patients, and only in 2 of 37 right hippocampal seizures of 2 patients.

**Conclusions:**

Seizure-related modulation of cardiac repolarization, but not of HR, appears to depend on the side of ictal activity, strengthening the hypothesis of asymmetric cerebral representation of cardiac function. The clinical relevance of this is unclear, but may indicate an increased risk of abnormal ictal QT prolongation in people with left mTLE.

## Introduction

Cardiac activity is dynamically modulated by the autonomic nervous system to ascertain sufficient blood and oxygen supply to all organs in response to various physiologic and pathophysiologic conditions. The major players consist of the sympathetic and parasympathetic branches which are regulated through cortical and subcortical neuronal networks including brain stem, insular cortex, amygdala, and hippocampus [1], [2]. Previous studies in human and animals have suggested an asymmetric representation of sympathetic and parasympathetic functions in the brain. For instance, electrical stimulation of the left insula or pharmacological inactivation of the right hemisphere predominantly led to a decrease in heart rate (HR), whereas right-sided stimulation or left-sided inactivation increased HR [3], [4]. Disturbances of cardiac repolarization have also been reported with seizures and other neurological conditions such as ischemic stroke [5]–[11]. Importantly, insults of the right hemisphere with insular involvement appear to be more frequently associated with prolongation of QT interval and potentially serious cardiac arrhythmias, supporting the notion of an asymmetrically represented cortical control of cardiac repolarization [5]–[7].

Taken together, cardiac function appears to be asymmetrically represented in the brain with potential clinical relevance according to the side of cerebral affection. To date, however, it is unclear whether transient disturbances of brain function e.g. by epileptic seizures have also a hemispheric-specific effect on cardiac function. Previous studies have produced controversial results, maybe due to variable interindividual patterns of cardiac regulation [11]–[13]. Based on the findings of three cases with seizures independently arising from both hemispheres in a given patient, we have recently hypothesized that periictal regulation of HR is individually lateralized [14], [15]. This hypothesis, however, was derived from three patients with scalp EEG recordings only, considerably limiting the strengths of these observations.

Here, we have asked whether seizure-related changes in HR and cardiac repolarization depend on the side of seizure-activity in people with medically refractory mesial temporal lobe epilepsy (mTLE) undergoing pre-surgical video-EEG telemetry using intracranial electrodes. We included patients with bilateral hippocampal depths electrodes and, to account for intrinsic differences in autonomic function and to allow intraindividual comparison, only those who had at least two seizures arising from each hippocampus independently from each other during video-EEG telemetry.

## Materials and Methods

### Patients and Inclusion Criteria

**Figure 1:**
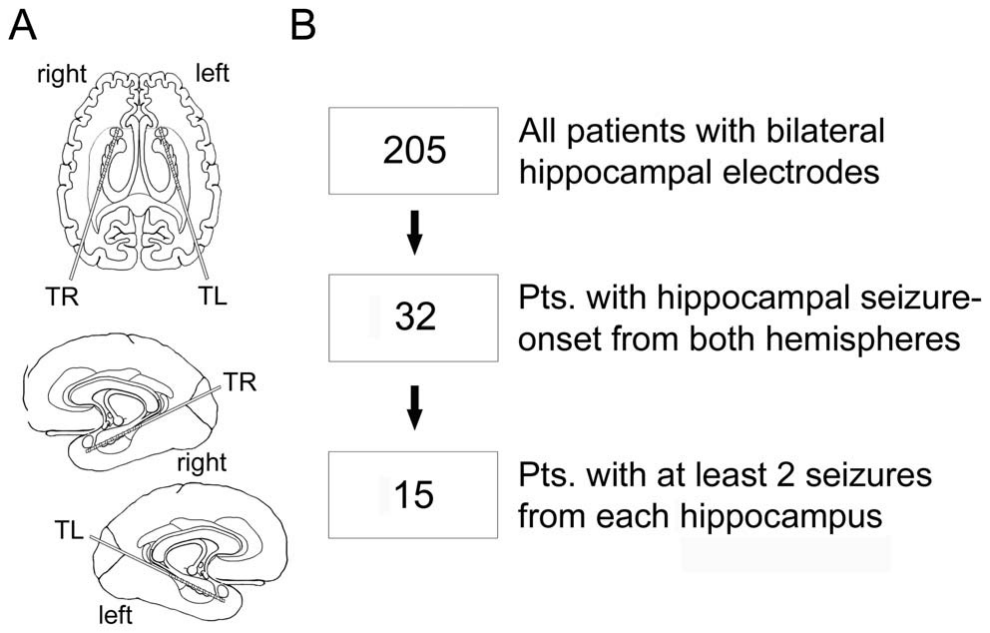
Implantation scheme and flowchart of patient selection. (A) Scheme of implantation of intracranial electrodes to assess hippocampal activity and (B) flowchart of selection and inclusion of patients.

We reviewed patients with medically refractory mTLE undergoing presurgical video-EEG monitoring during a 12 years period from January 2000 to December 2011 in the Department of Epileptology at the University Hospital Bonn (Germany). This study is a retrospective audit of EEG and ECG data which have been collected during standard clinical procedures, and has been approved as such by the local medical ethics committee (“Ethikkommission an der Medizinischen Fakultaet der Rheinischen Friedrich-Wilhelms-Universitaet Bonn”). Informed patient consent was not required because of the retrospective design and the anonymization of patient-related data for analysis and publication. Inclusion criteria were presence of bilateral hippocampal depth electrodes and the occurrence of at least two seizures with independent onset from each hippocampus. Two to 4 seizures per hemisphere per patient were analyzed. Seizures were selected according to the side of seizure-onset and the interpretability of simultaneous ECG traces. If more than 2–4 seizures per patient and hemisphere were eligible for analysis, seizures were selected in chronological order of occurrence. The correct position of the electrodes was controlled with the help of MRI after implantation. In 13 patients, hippocampal depths electrodes (Ad-tech®, Racine, WI, USA) were implanted via a posterior approach as displayed in figure 1A and described in table 1. In one patient, the amygdala and the body of the hippocampus were covered by two depths electrodes implanted via a lateral approach, and in the remaining patient, 5 depths electrodes via a lateral approach were implanted on each hemisphere and covered the amygdala, hippocampus (head, body and tail) as well as anterior entorhinal cortex and posterior parahippocampal gyrus.

**Table 1:** Clinical characteristics of patients.

Patientno.	Sex	Age*/Epilepsyduration*/handedness	MRI finding	Surgery	Intracranial electrodes	FU§/Outcome#
48	M	32/32/R	Bilat. HS	No	Hipp: 1 depth electrode (10 c.) on each side from posteriorExHipp: None	n.a.
105	M	39/3/R	Bilat. HS R>L	No	Hipp: 1 depth electrode (10 c.) on each side from posteriorExHipp: None	n.a.
111	M	28/23/L	HS L	SAHE L	Hipp: 1 depth electrode (10 c.) on each side from posteriorExHipp: 1 temporo-lateral (16 c.) and 2 temporo-basal (4 c.)strip electrodes on left side	48/I
112	F	24/4/R	Bilat. HS L>R	No	Hipp: 1 depth electrode (10 c.) on each side from posteriorExHipp: 1 temporo-lateral (4 c.) and 2 temporo-basal (4 c.)strip electrodes on each side	n.a.
119	F	55/33/R	Bilat. HS L>R	SAHE R	Hipp: 1 depth electrode (10 c.) on each side from posteriorExHipp: 1 temporo-lateral (6 c.) and 2 temporo-basal (4 c.)strip electrodes on each side	53/II
127	M	45/39/L	None	No	Hipp: 1 depth electrode (10 c.) on each side from posteriorExHipp: 1 temporo-lateral (4 c.) and 2 temporo-basal (4 c.)strip electrodes on each side	n.a.
132	F	47/7/R	Bilat. HS	No	Hipp: 1 depth electrode (10 c.) on each side from posteriorExHipp: None	n.a.
135	F	46/33/R	HS R	SAHE R	Hipp: 1 depth electrode (10 c.) on each side from posteriorExHipp: 1 temporo-lateral (6 c.) and 2 temporo-basal (4 c.)strip electrodes on each side	18/II
143	M	22/15/L	None	SAHE L	Hipp: 1 depth electrode (10 c.) on each side from posteriorExHipp: 2 temporo-basal (4 c.) strip electrodes on each side1 temporo-lateral strip electrode (6 c.) on right side and1 grid electrode (32 c.) on left side covering Wernicke’s area	24/I
144	F	35/30/R	Bilat. HS L>R	No	Hipp: 1 depth electrode (10 c.) on each side from posteriorExHipp: None	n.a.
160	F	31/30/R	Bilat. HS	TL-resection incl. AHE L	Hipp: 1 depth electrode (10 c.) on each side from posteriorExHipp: None	No FU-visit
182	F	34/14/R	Bilat. HS L>R	No	Hipp: 1 depth electrode (10 c.) on each side from posteriorExHipp: None	n.a.
184	F	28/25/L	HS R	No	Hipp: 2 depth electrodes (8 c.) on each side from lateralExHipp: 2 temporo-basal (4 c.) strip electrodes on each side	n.a.
187	F	31/29/R	Bilat. HS	No	Hipp: 1 depth electrode (10 c.) on each side from posteriorExHipp: 1 temporo-lateral (6 c.) and 2 temporo-basal (4 c.)strip electrodes on each side	n.a.
202	M	28/15/R	HS L	No	Hipp: 5 depths electrodes (10 c.) on each side from lateralExHipp: 2 frontal strip electrodes (8 c.) on each side	n.a.

* At telemetry.

§ follow-up in months.

# according to Engel classification.

c, electrode contacts; ExHipp, extrahippocampal; Hipp, hippocampal; HS, hipppocampal sclerosis; L, left; n.a., not applicable; R, right; SAHE, selective amygdala-hippocampectomie; TL, temporal lobe.

### Presurgical Evaluation

Standard presurgical assessment included cerebral MRI (1.5 or 3 Tesla), non-invasive video-EEG telemetry using scalp EEG (10–20 system with additional temporal electrodes) prior to invasive video-EEG telemetry and neuropsychological testing. Video-EEG telemetry is the simultaneous acquisition of a video film (to observe the clinical symptoms of the patients) and EEG recordings (to assess the regional onset and propagation of the seizure activity) in order to correlate the patients’ behaviour with the seizure activity. In all included patients, invasive video-EEG monitoring was performed with hippocampal depth electrodes (see above) and in some patients with additional neocortical subdural strip or grid electrodes (table 1). All electrodes were placed in dependence of MRI findings, prior findings in scalp EEG recordings and seizure semiology.

### EEG Recordings

EEG data acquisition was performed with a Stellate Harmonie digital video-EEG system (Version 5.4, Schwarzer GmbH/Natus, Germany) using up to 128 channels, a 200 Hz sampling rate and a 16 bit analogue-to-digital converter. Data were band pass filtered between 0.016 and 70 Hz. We determined the timepoint and localization of seizure-onset according to the intracranial EEG recordings.

### Periictal One-lead ECG Recordings

A modified lead-I ECG (adhesive electrodes placed below the clavicles of either side) was recorded simultaneously with EEG. RR intervals were determined manually at different timepoints (1 min before EEG seizure-onset, during unilateral hippocampal activity where RR was shortest in order to “normalize” the data; 1 min after seizure cessation). QT intervals were manually measured from the start of the QRS complex to the end of the T wave (defined by the intersection with the isoelectric line). QT and preceding RR intervals were determined from 3–5 successive ECG complexes and QT intervals were corrected for variable RR intervals. All correction formulas tend to over- or underestimate QTc [16]. To minimize errors due to correction bias and as a sensitivity analysis, we have used four established formulas as follows:

(1) Bazett: QTc = QT/RR^1/2^
(2) Fridericia: QTc = QT/RR^1/3^
(3) Framingham: QTc = QT +0.154×(1-RR)
(4) Hodges: QTc = QT +1.75×(HR –60)

In formulas (1)–(3), QTc, QT and RR interval values are expressed in seconds. In (4), QTc and QT intervals are in milliseconds and HR is in beats per minute. To reduce a bias error of putatively pathologic QTc intervals (due to higher HR), we have used modified normal limits for QTc intervals as proposed by Luo and colleagues (see table S1) [17].

### Statistics

Statistical differences of HR and QTc values between seizures with left- and right-sided ictal activity were assessed pairwisely using a mixed linear regression model adjusted for variable seizure numbers per patient and individual patient effects (STATA12 software, StataCorp LP, TX, U.S.A.). P-values <0.05 were regarded as statistically significant, based on the null hypothesis that there is no hemispheric side effect on (i) ictal HR and (ii) QT intervals. All data are given as means ± S.E.M.

## Results

Fifteen (9 women; age 35.0±2.5 years) of 205 patients with medically refractory mTLE fulfilled the inclusion criteria (Figure 1B). Thirteen patients had signs of hippocampal sclerosis on cerebral MRI and 2 patients had no detectable MRI abnormalities (Table 1). None of the patients had been diagnosed with a cardio-pulmonary disease.

**Figure 2:**
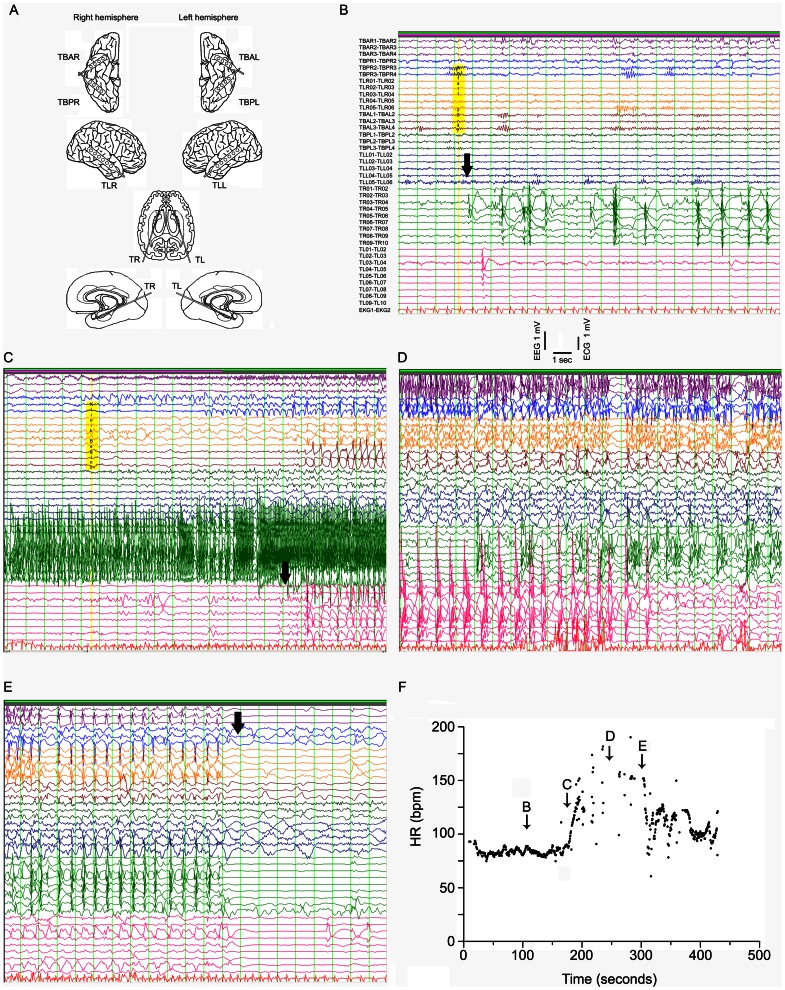
Example of original EEG- and ECG-traces during a focal seizure with right-sided hippocampal onset. (A) Implantation scheme of intracranial electrodes (patient no. 119). (B–E) EEG-traces in bipolar montage (localization as given in panel A, the lower numbers apply to the contacts opposite to the cable outlet of the respective strip or depths electrodes) and ECG-traces (last trace, labeled as EKG1-EKG2, represents derivation Einthoven II with inverted polarity). The time period of the recordings is indicated in panel F. (B) Arrow indicates seizure-onset in the right hippocampus. (C) The arrow indicates onset of ictal activity in the left hippocampus. (D) Note the compromised ECG trace due to movement artifacts of the patient. (E) The arrow indicates the abrupt termination of seizure activity. (F) Time course of HR during this focal seizure with impaired responsiveness and complex automatisms. The arrows indicate the time periods from which example panels B–E have been selected. Note the missing values after propagation of ictal activity to the left hemisphere (time period between arrows “C” and “E”).

**Figure 3:**
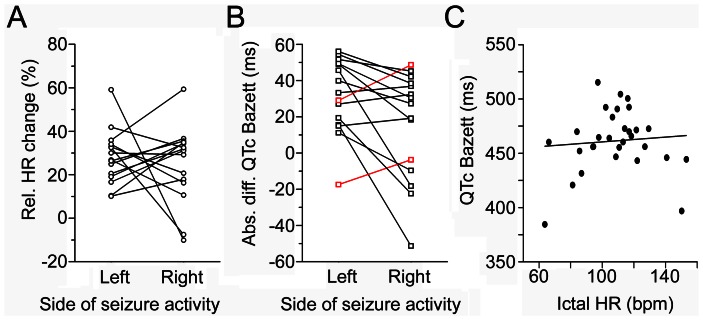
Plot of HR and QTc changes per patient. (A) Relative ictal HR changes and (B) absolute QTc differences using Bazett’s formula were plotted separately for each patient and side of seizure activity. Corresponding data pairs from each patient were connected with a line. Note that only in two patients, QTc increased by more than 10 ms during right hippocampal seizures as compared to left hippocampal seizures (B, highlighted in red). (C) Individual QTc values (Bazett) did not correlate with corresponding absolute ictal heart rates (linear regression, p = 0.67). Examples were illustrated using Bazett’s formula, as this correction formula is known to overestimate corrected QT values, so that a potential artificial bias, if present, should be clearly visible.

**Figure 4:**
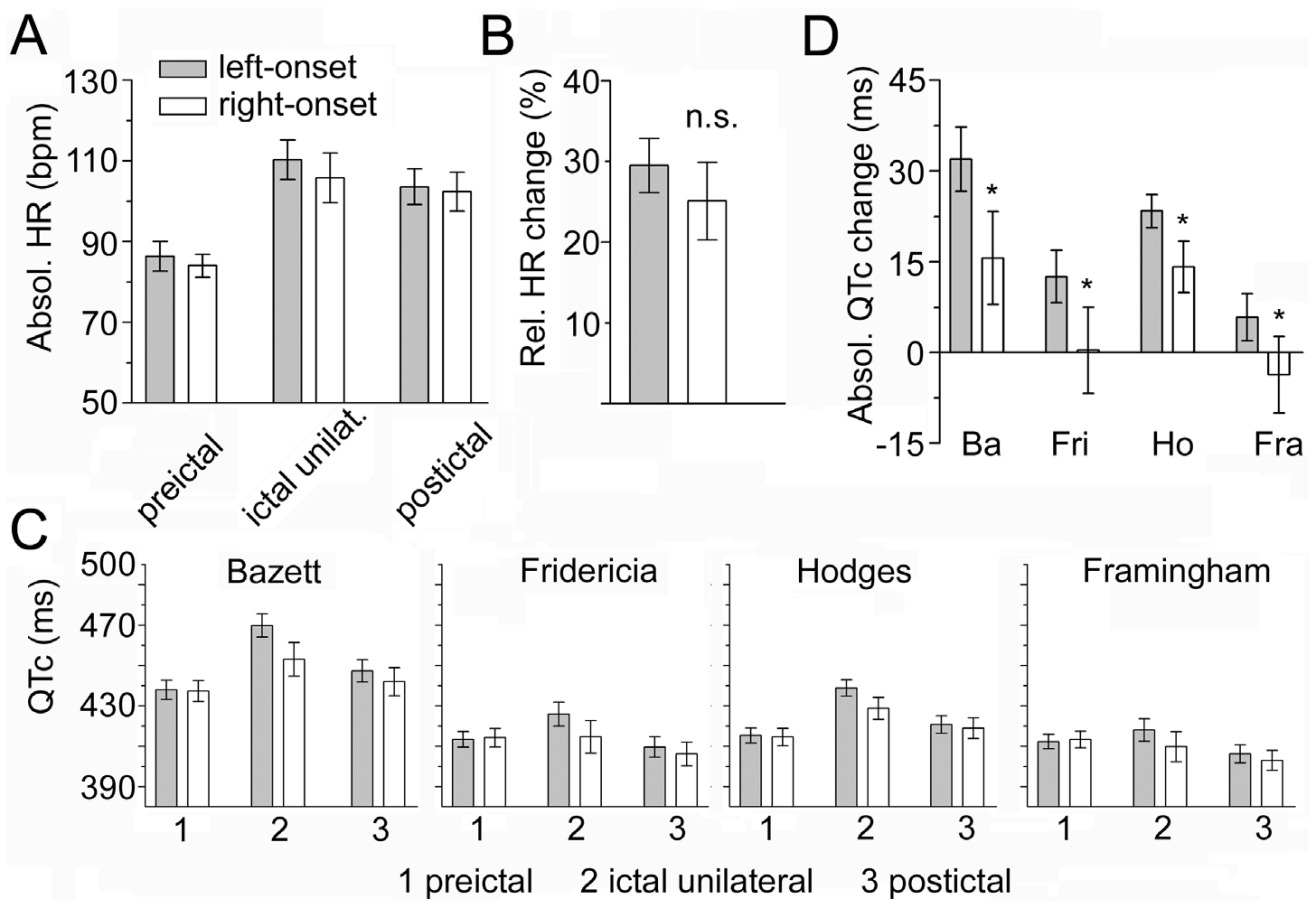
HR and QTc increase with ictal activity, whereupon modulation of QTc, but not of HR, is asymmetrically lateralized. (A) Absolute HR at different timepoints from all patients was averaged (based on a mean HR per timepoint and side of seizure-onset per patient). Paired data for right- (white bars) and left-hippocampal onset (grey bars) were available from all 15 patients at all timepoints. (B) Relative HR changes from all patients were averaged with no significant difference of ictal modulation of HR between left- and right-onset seizures. (C) QT intervals corrected with all four formulas (grey bars, left-hippocampal seizures; white bars, right-hippocampal seizures) were plotted versus three timepoints (1, preictal; 2, unilateral ictal activity; 3, postictal). (D) The absolute ictal changes of QT intervals using all four correction formulas (Ba, Bazett; Fri, Fridericia; Ho, Hodges; Fra, Framingham) were separately plotted for left- (grey bars) and right-hippocampal seizures (white bars). QT lengthening was significantly greater during left-hippocampal activity as assessed with all 4 correction formulas, suggesting an asymmetric ictal modulation of cardiac repolarization. All data expressed as mean±S.E.M.

A total of 82 seizures with unilateral hippocampal onset were analyzed (Table 2). A representative example of original EEG- and ECG tracings is given in figure 2. Overall, HR increased by ∼30% during seizure activity confined to the hippocampus without significant differences between left- and right-sided seizures (p = 0.411; Figure 3A; 4A,B).

**Table 2:** Seizure characteristics.

Patient no.	Side of hippocampal seizure activity	Seizure types	Duration* (s)
48	Left	CPS; SGTCS; CPS	180; 184; 113
	Right	CPS; CPS	121; 154
105	Left	SPS; SPS; SPS	118; 132; 137
	Right	SPS; SPS	75; 254
111	Left	CPS; CPS; CPS; CPS	79; 120; 71; 60
	Right	CPS; CPS	37; 38
112	Left	CPS; CPS; CPS	66; 95; 73
	Right	CPS; CPS	87; 47
119	Left	SCP; SCP; SCP	65; 68; 144
	Right	CPS; CPS; CPS	127; 200; 100
127	Left	CPS; CPS; CPS	130; 71;180
	Right	CPS; CPS; CPS	282; 96; 195
132	Left	CPS; CPS;CPS	61; 87; 91
	Right	SPS; SPS; CPS	78; 64; 132
135	Left	SPS; SPS	42; 47
	Right	CPS; CPS; SPS	66; 64; 255
143	Left	CPS; CPS; CPS	102; 115; 67
	Right	SGTCS; CPS	128; 77
144	Left	SPS; CPS	163; 243
	Right	SPS; SPS; SPS	141; 20; 289
160	Left	SPS/CPS#; CPS	101; 120
	Right	CPS; CPS	69; 137
182	Left	CPS; SGTCS; CPS	102; 118; 104
	Right	SCP; SCP; SGTCS; SGTCS	30; 35; 191; 196
184	Left	SPS/CPS#; CPS; SPS	60; 111; 28
	Right	SGTCS; CPS; SCP; SPS	273; 188; 44; 80
187	Left	CPS; CPS;CPS	391; 173; 193
	Right	SCP; SCP; SCP	118; 86; 65
202	Left	CPS; SGTCS	134; 147
	Right	CPS; CPS	188; 143

* According to EEG pattern.

# Consciousness not tested.

CPS, complex-partial seizures; SGTCS, secondarily generalized tonic-clonic seizure; SCP, subclinical EEG pattern (no objective clinical signs apart from alterations of cardiac activity and with or without testing); SPS, simple partial seizure.

QTc intervals, however, were prolonged to a significantly greater extent with ongoing hippocampal seizures on the left side after adjustment with all 4 correction formulas (p-values: Bazett 0.016; Fridericia 0.027; Hodges 0.038; Framingham 0.041; Figure 3B; 4C,D). Importantly, absolute QTc values were not correlated with the absolute HR, suggesting that there was no major bias introduced by using correction formulas (Figure 3C). Abnormal QTc prolongation according to all 4 correction formulas occurred in 7 of 41 left hippocampal seizures of 4 patients, and only in 2 of 37 right hippocampal seizures of 2 patients (Table 3). Absolute QTc shortening below 10 ms according to all 4 correction formulas occurred in 5 of 37 right hippocampal seizures of 4 patients, and in 2 left hippocampal seizures of 2 patients. Abnormal QTc shortening occurred only in 1 right hippocampal seizure (Table 3).

**Table 3:** Summary of seizure-related QT alterations.

Abnormal QTc prolongation in all 4 formulas according to Luo et al. 2004*		
	Left-hippocampal seizures (n = 41)	Right-hippocampal seizures (n = 37)
Seizures (no./%)	**7/17.1%**	2/5.4%
Patients (no./%)	**4/26.7%**	2/13.3%
Abnormal QTc prolongation above 500 ms according to Bazett’s formula		
	Left-hippocampal seizures (n = 41)	Right-hippocampal seizures (n = 37)
Seizures (no.)	**5/12.2%**	2/5.4%
Patients (no.)	**3/20%**	2/13.3%
Abnormal QTc shortening according to Luo et al. 2004* (using Bazett, Fridericia, Framingham)		
	Left-hippocampal seizures (n = 41)	Right-hippocampal seizures (n = 37)
Seizures (no.)	0/0%	1/2.7%
Patients (no.)	0/0%	1/6.7%
QTc shortening ≤ −10 ms in all 4 formulas		
	Left-hippocampal seizures (n = 41)	Right-hippocampal seizures (n = 37)
Seizures (no.)	2/4.9%	**5/13.5%**
Patients (no.)	2/13.3%	**4/26.7%**

* Luo S, Michler K, Johnston P, Macfarlane PW. A comparison of commonly used QT correction formulae: the effect of heart rate on the QTc of normal ECGs. J Electrocardiol. 2004;37 Suppl: 81–90 (see table S1). In 5 of the 82 included seizures, ictal QT intervals could not been reliably analyzed.

No potentially serious periictal cardiac arrhythmias were observed, but only 6 short episodes of ictal or postictal pronounced sinus arrhythmia without consistent association with seizure-onset from one hippocampus.

## Discussion

Our study suggests asymmetric seizure-related modulation of cardiac repolarization. There are controversial data on lateralized cerebral representation of cardiac function. Most studies have assessed HR in response to iatrogenic interventions. Studies using pharmacological inactivation of both hemispheres via intracarotid amobarbital injection have produced conflicting data [4], [18]–[20]. Nevertheless, an increase of HR following inactivation of the left hemisphere was observed in all four studies, whereas HR displayed an increase [18], [19], a decrease [4] or no significant change [20] upon right-sided inactivation, supporting at least the notion that cortical networks tonically modulate sympathetic and parasympathetic activity. The reasons for this controversy are unclear, but a variable representation and function of the right hemisphere, a region-specific excitatory or inhibitory modulation and different cerebral epileptogenic lesions in these studies may play a role. In another study, electrical stimulation of the insula was performed in 5 patients and shown that a decrease of HR occurred more frequently upon stimulation of the left insula, whereas increase in HR was more often elicited by stimulation of the right insula, suggesting a differential distribution of cardiovascular networks on both hemispheres [3].

In contrast to these studies, we have investigated ECG features in response to spontaneously occurring seizures. It is not surprising that seizure activity within the hippocampus has effects on cardiac function, as a number of anatomical pathways connect the hippocampus and the amygdala with other brain regions known to modulate heart activity (e.g. with the insula and cingulate gyrus or via the Papez circuit with thalamic and hypothalamic projections to the brain stem) [1], [21]. Capitalizing on a pair-wise comparison, we have found that cardiac repolarization, but not HR, is differentially modulated during limited hippocampal seizure activity of the left and right hemisphere. The lack of laterality in HR regulation is in line with a number of previous studies which have shown that e.g. ictal HR was not correlated with the side of seizure activity and that ictal bradycardia appeared to be associated with bilateral spread of ictal activity, but not with lateralized seizure activity [11], [13], [22].

The asymmetric cerebral control of cardiac repolarization is supported by a number of human and animal studies investigating QT intervals following neurological injuries [5]–[7], [23]. Importantly, Critchley and co-workers have investigated the cerebral areas involved in the modulation of cardiac activity during mental and physical stress in people without apparent affection of the brain [24]. To that end, they have recorded regional blood flow (as assessed by H_2_^15^O-PET scans) and simultaneously recorded ECG features during a subtraction task with or without time restriction (mental stress paradigm) and isometric handgrip squeeze under specific conditions (physical stress paradigm). The major findings are that proarrhythmic abnormalities of cardiac repolarization were positively correlated with an asymmetric increase in regional blood flow in the midbrain on the right side, and, at least in one feature of QT abnormality, also in the right parahippocampal gyrus, supporting the notion of an asymmetric representation of the autonomic control of cardiac repolarization including mesiotemporal brain structures. These findings may explain our observation that seizure-related modulation of cardiac repolarization appears to be asymmetrically localized in people with mesial TLE. Accordingly, right- and left-sided hippocampal seizure activity may induce an asymmetric sympathetic modulation of cardiac repolarization via mesiotemporal networks (parahippocampal gyrus) and midbrain networks (via projections to the brain stem). Interestingly, QTc intervals shortened again after the propagation of seizure activity to the contralateral hemisphere, (probably linked to bilateral activation of involved neuronal mesiotemporal and midbrain networks) and returned to baseline postictally (figure S1).

Whereas the above cited studies include patients or animals with a structural lesion due to ischemic stroke or in people during physiologic stress tasks (and without known brain diseases), we have assessed the effects of ongoing abnormal seizure activity in a limited neuronal network. It is tempting to speculate that, in analogy to *positive* clinical signs such as motor activity or somatosensory sensations, seizure activity leads to activation of autonomic cardiac networks. It remains, however, unclear whether pathologic ictal activity has excitatory or inhibitory (disrupting) effects on autonomic networks regulating cardiac repolarization.

### Clinical relevance of asymmetric seizure-related modulation of cardiac repolarization

Cardiac repolarization was assessed as QT intervals corrected with four established formulas to account for selective bias using e.g. Bazett’s formula only (which is known to overestimate corrected QT intervals). QTc was not correlated with absolute ictal HR (Figure 3C), suggesting that a major bias by the correction procedure is unlikely. In our study, QTc intervals were lengthened to a greater extent during left hippocampal seizure activity, in average by more than 30 ms (range −17 ms to 56 ms) during left- and by about 15 ms (range −51 ms to 48 ms) during right-sided hippocampal activity, strengthening the hypothesis of side-dependent regulation of cardiac function. This averaged difference in QTc intervals appears to be subtle. At the level of individual seizures, however, abnormal QTc prolongation above normal upper limits was more frequently observed with left hippocampal seizures, whereas abnormal QTc shortening below normal limits was noted in one right-sided seizure only (table 3). The clinical relevance of these findings is not clear at present. They may indicate an increased risk of abnormal ictal QT prolongation in people with left mTLE. This could be especially relevant in the presence of drugs interfering with cardiac repolarization [25]. Seizure-related abnormal prolongation and shortening of QT intervals have recently been described [8]–[11], could facilitate onset of ventricular tachyarrhythmia and thereby contribute to the pathophysiology of sudden unexpected death in epilepsy (SUDEP) [26], [27]. One established risk factor for SUDEP is the presence of generalized convulsive seizures (GCS), potentially due to GCS-related cardiorespiratory dysfunction [27]. It is of note that abnormal QTc shortening predominantly occurs with GCS [10], providing a possible link between seizure-related pathologic cardiac repolarization and fatal ventricular tachyarrhythmia. In some witnessed SUDEP cases, however, GCS have not been reported or sudden death has occurred in association with an epileptic aura [28]. In this context, our findings may be of particular importance: We have detected seizure-related abnormal QT alterations during ictal activity confined to one hippocampus and temporal lobe, suggesting that even seizures without extended involvement of the brain (such as auras) and without generalized convulsions may bear the risk of sudden cardiac death as one cause for SUDEP.

### Study Limitations

This is a retrospective study capitalizing on intracranial EEG recordings. Implantation of electrodes was performed according to an *a-priori* hypothesis of seizure-onset zone based on electro-clinical and MRI findings during non-invasive presurgical assessment with the ultimate goal of using as few electrodes as possible (to minimize potential risks and complications). Therefore, the number of implanted electrodes is limited with a consecutive spatial sampling bias. Ictal activity tends to spread from the hippocampus to lateral and basal parts of the temporal lobe and the insula of the same hemisphere, and later during the seizure, to the contralateral hemisphere as well [29]. In this context, it is important to note that ictal HR appears to increase gradually with regional spreading of ictal activity [30]. Thus, we cannot rule out that at the timepoint where we have assessed RR and QT intervals, ictal activity within the hippocampus has propagated to other, neocortical ipsilateral or contralateral regions of the brain. We have analyzed HR changes in a subgroup of our patients who had additional strip and grid electrodes (figure S2) and found that the extent of relative HR changes (as a rough measure of ictal spread) was in the same range as compared to the data of all patients, suggesting limited seizure activity within the ipsilateral temporal lobe. Another weakness of our study is that we have only analyzed ECG data at arbitrarily selected timepoints (e.g. preictal and postictal values were assessed at a given time interval before and after the seizure, whereas ictal HR was determined where RR intervals were shortest and where 3 consecutive QRS intervals allowed manual measurement of both RR and QT intervals). Thus, we predominantly describe “snapshots” of cardiac activity during seizures, but do not have information on the time course of seizure-related cardiac regulation. It would be interesting to know the entire dynamics of HR and cardiac repolarization during seizures arising from the left and right hemisphere. This question, however, has not been directly addressed in our study. Another important methodical limitation of our study stems from the fact that correction of QT intervals is very complex. Even under steady-state conditions, the relationship between QT and RR intervals is not linear. For instance, when cycle length varies, QT intervals are influenced by changes in the preceding cycle length and by preceding interbeat intervals [31]. These adaptations may take 30 to 120 seconds [31]. Seizure-related HR changes are variable throughout the course of epileptic seizures (Figure 2), e.g. HR can steadily increase and stay on a certain level for some time (e.g. 30 seconds), and then return to baseline again, or display rather rapid alterations with increases, decreases and increase again or vice-versa [15]. Furthermore, seizures usually last for about 30 to 90 seconds. Thus, if the “true” QT values require many seconds or several minutes to be set, our data on corrected QT intervals may be of limited clinical relevance. These difficulties cannot be overcome and are inherent to the paroxysmal nature of epileptic seizures (which represent transient disturbances of the brain activity with a limited duration only). Our study, however, was designed to tackle the question whether there is a difference between left- and right sided seizures within a given patient across several seizures from each side. This means that the inherent limitations apply at least to both conditions (left versus right), which strengthens, to some extent, the relevance of our findings.

A further weakness of our study is the lack of information on periictal respiratory function in our patients, as seizure-related hypoxemia increases the likelihood of both QTc prolongation and shortening [32]. However, ictal apnea appears to be correlated with spread of seizure-activity to the contralateral hemisphere, and not with a particular side of seizure-onset or seizure-lateralization [33], supporting our conclusions on the link between ictal modulation of autonomic networks and alteration of cardiac repolarization. In addition, anticonvulsant drugs such as rufinamide and primidone have been reported to modulate cardiac repolarization [25], [34]. Our patients, however, have not been on these drugs during video-EEG telemetry. The strength and the additional value of this study is the comparison of seizures arising from both hippocampi within the same patients, thereby controlling for a great portion of intra-individual variability of cardiovascular and autonomic features and allowing pair-wise analysis of HR and QT intervals. In addition, we have investigated a relatively homogenous study population which may further reduce errors due to lesion type and cerebral localization of the focus as a potential confounder. Importantly, the presence of hippocampal sclerosis *per se* has no measurable effect on the cardiovascular autonomic properties as compared to people with focal epilepsy without hippocampal sclerosis [35]. Using this conservative approach, our study population has undergone a strong selection with a limited final sample size, thereby weakening the statistical power of our study. Despite this high selection, comparison of corrected QT intervals has reached statistically significant differences, strengthening the hypothesis of an asymmetric representation of autonomic networks involved in the regulation of cardiac repolarization.

## Supporting Information

Figure S1 **QTc decreases after propagation to the contralateral hemisphere.**
(DOCX)

Figure S2 **Extent of ictal HR changes correlates with spatial spread, but not with duration of ictal activity.**
(DOCX)

Table S1 **Upper and lower normal limits of normal QTc for 4 correction formulas.**
(DOCX)
