# Supplementary material for: Ictal Modulation of Cardiac Repolarization, but Not of Heart Rate, Is Lateralized in Mesial Temporal Lobe Epilepsy
Source: PLoS One. 2013 May 31;8(5):e64765. doi: 10.1371/journal.pone.0064765 (PMC3669418; doi:10.1371/journal.pone.0064765)
Supplement: Figure S1 — QTc decreases after propagation to the contralateral hemisphere. (DOCX) [file pone.0064765.s001.docx]

**Figure S1. QTc decreases after propagation to the contralateral hemisphere.**

Absolute QTc changes (using Fridericia’s formula) were plotted during unilateral hippocampal activity, after propagation to the contralateral hemisphere (bilateral hippocampal seizure activity) and after seizure cessation (postictal) for each hemisphere. QTc increased with unilateral seizure activity and decreased after propagation to the contralateral hemisphere, with a more pronounced decrease with seizures displaying a right-onset. Data available for 11 patients and left-onset seizures, and 9 patients and right-onset seizures.
