# Supplementary material for: Ictal Modulation of Cardiac Repolarization, but Not of Heart Rate, Is Lateralized in Mesial Temporal Lobe Epilepsy
Source: PLoS One. 2013 May 31;8(5):e64765. doi: 10.1371/journal.pone.0064765 (PMC3669418; doi:10.1371/journal.pone.0064765)
Supplement: Figure S2 — Extent of ictal HR changes correlates with spatial spread, but not with duration of ictal activity. (DOCX) [file pone.0064765.s002.docx]

**Supplementary information**

A methodical weakness of our study is the fact that in 6 of our 15 patients, data interpretation is limited due to a spatial sampling bias, as those patients had bilateral hippocampal electrodes only (Figure 1A, table 1). In 7 patients, however, depths electrodes were placed within the hippocampus as well as additional strip and grid electrodes on lateral and basal parts of the temporal lobe on both sides, allowing correlation of HR change and spatial spread of ictal activity. ECG signals were analyzed 1 min before seizure onset, after unilateral hippocampal onset (at the timepoint where HR was highest and before spread to other regions than ipsilateral hippocampus), after spread to ipsilateral neocortical areas (regional) and after spread to contralateral hemisphere (hippocampus or neocortical electrodes). In line with previous reports [Epstein et al., 1992], we have observed that the extent of ictal HR changes depends on the degree of spatial spread, but not on the duration of ictal activity (supplementary figure).


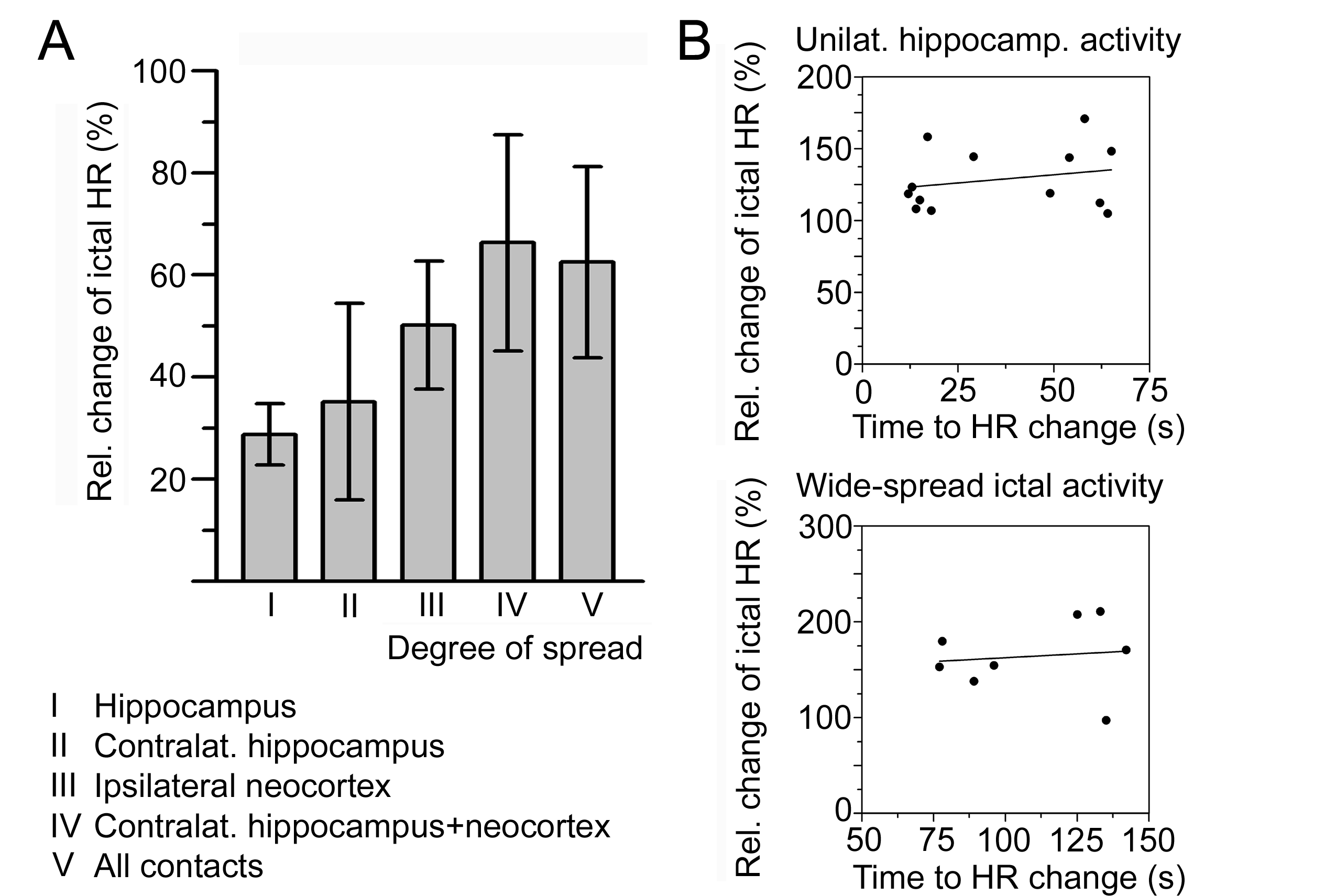


**Figure S2. Extent of ictal HR changes correlates with spatial spread, but not with duration of ictal activity.**

(A) HR changes relative to preictal values were plotted versus the spatial spread (from focal hippocampal activity to wide-spread ictal activity covering all available intracranial contacts; indicated as I-V). Two seizures per patient (only of those 7 patients who had hippocampal depths electrodes and additional strip or grid electrodes covering the temporal lobe on both sided) were included (one with right, one with left-sided hippocampal onset). Final number of included data points (from left bar to right bar): 14, 4, 5, 3 and 5. Data expressed as mean±S.E.M. (B) Relative change of ictal HR was plotted versus the time interval between EEG-onset and timepoint of HR assessment at two ictal phases: during unilateral hippocampal activity (upper panel; linear regression p=0.44) and during wide-spread ictal activity (with additional ictal activity in the contralateral hippocampus and neocortex or in all available contacts; lower panel, p=0.79).
