# Supplementary material for: Ictal Modulation of Cardiac Repolarization, but Not of Heart Rate, Is Lateralized in Mesial Temporal Lobe Epilepsy
Source: PLoS One. 2013 May 31;8(5):e64765. doi: 10.1371/journal.pone.0064765 (PMC3669418; doi:10.1371/journal.pone.0064765)
Supplement: Table S1 — Upper and lower normal limits of normal QTc for 4 correction formulas. (DOCX) [file pone.0064765.s003.docx]

| **Table S1. Upper and lower normal limits of normal QTc for 4 correction formulas.**  **Adapted from Luo et al., (2004) A comparison of commonly used QT correction formulae: the effect of heart rate on the QTc of normal ECGs. J Electrocardiol 37 Suppl: 81-90.** | | | | | |
| --- | --- | --- | --- | --- | --- |
|  | **Upper normal limits (98%, in ms)** | | | | |
| **Gender** | **HR (bpm)** | **QTcB** | **QTcFri** | **QTcFra** | **QTcH** |
| Both | All HR | 483 | 460 | 457 | 457 |
|  | HR < 60 | 454 | 459 | 459 | 466 |
|  | HR 60 to 99 | 483 | 461 | 458 | 456 |
|  | HR > 99 | 492 | 445 | 436 | 451 |
| Male | All HR | 480 | 457 | 454 | 454 |
|  | HR < 60 | 450 | 455 | 455 | 465 |
|  | HR 60 to 99 | 480 | 457 | 454 | 452 |
|  | HR > 99 | 490 | 445 | 436 | 450 |
| Female | All HR | 486 | 463 | 461 | 460 |
|  | HR < 60 | 460 | 463 | 463 | 470 |
|  | HR 60 to 99 | 486 | 465 | 462 | 459 |
|  | HR > 99 | 492 | 448 | 434 | 452 |
|  | | | | | |
|  | **Lower normal limits (2%, in ms)** | | | | |
| **Gender** | **HR (bpm)** | **QTcB** | **QTcFri** | **QTcFra** | **QTcH** |
| Both | All HR | 378 | 365 | 368 | 372 |
|  | HR < 60 | 363 | 372 | 367 | 375 |
|  | HR 60 to 99 | 382 | 370 | 374 | 372 |
|  | HR > 99 | 380 | 343 | 350 | 371 |
| Male | All HR | 374 | 361 | 364 | 369 |
|  | HR < 60 | 361 | 368 | 363 | 369 |
|  | HR 60 to 99 | 379 | 368 | 372 | 369 |
|  | HR > 99 | 378 | 341 | 348 | 369 |
| Female | All HR | 386 | 369 | 372 | 377 |
|  | HR < 60 | 372 | 381 | 377 | 386 |
|  | HR 60 to 99 | 388 | 374 | 378 | 376 |
|  | HR > 99 | 389 | 350 | 354 | 374 |
|  |  |  |  |  |  |
